# 

**Published:** 1881-01

**Authors:** 


### THE BISTOURY:

Thad S. Up de Graff, M. D., Editor & Proprietor.

### *CONTENTS FOR JANUARY, 1881.*

**CONTRIBUTIONS.** PAGE.

About Livers.............................Ben. H. Pratt,.......................................... 310
The Law's Charlatanry,,.....................Ausburn Towner..................................... 313
Words.............................J. J. Keyes............................................ 316
Local Microscopical Societies.................Geo. E. Blackham, M. D., F. R. M. S.......................... 317
Labor-Saving.............................Thos. K. Beecher........................................ 319

**MEDICAL ITEMS AND NEWS.**
Jamaica Dogwood—Sore Nipples—Balsam Copaiba Emulsion—Asthma—Rectal Alimentation—Toothache—Hemorrhoids—Chian Turpentine Emulsion—Administration of Iodide of Potassium—Champhor and Chloral—Diphtheria—Digestive Derangements—Diphtheria—Poisoning—Vehicle for Salicylic Acid—Nitro-glycerine—Itch—Castor-oil Emulsion—Onions in Consumption—Filling for Teeth—Antiseptic Dressings—Anthelmintic Action of Kameela—New Method of Arresting Gonorrhœa—Diphtheria—Sweating of the Feet—Hospital Practice..321 325

**OUR CORRESPONDENCE** Two Letters with answers.............................................326

**EDITORIAL.**
Free Schools................................................................ 327

**CHIT-CHAT.**
New Type—Bound Volumes—Crowded Out—American Society of Microscopists—"Satisfactory Settlement"—The Klan—Sara Bernhardt—The Irish Question—Cremation at Warren, Pa.—The Beecher Supper—End of Volume XVI—Rochester Opticians—A Skating Rink Needed—Christmas Presents—The Bistoury........329 to 331

